# Organic Zeolite Analogues Based on Multi-Component Liquid Crystals: Recognition and Transformation of Molecules within Constrained Environments

**DOI:** 10.3390/ma4010183

**Published:** 2011-01-11

**Authors:** Yasuhiro Ishida

**Affiliations:** 1 RIKEN, Advanced Science Institute, 2-1 Hirosawa, Wako, Saitama 351-0198, Japan; 2 PRESTO, Japan Science and Technology Agency, 4-1-8 Honcho, Kawaguchi, Saitama 332-0012, Japan; 3 Department of Chemistry and Biotechnology, Graduate School of Engineering, The University of Tokyo, Hongo, Bunkyo-ku, Tokyo 113-8656, Japan

**Keywords:** liquid crystals, supramolecular chemistry, cross-linking, template reactions, host-guest chemistry, chiral recognition

## Abstract

In liquid crystals (LCs), molecules are confined in peculiar environments, where ordered alignment and certain mobility are realized at the same time. Considering these characteristics, the idea of “controlling molecular events within LC media” seems reasonable. As a suitable system for investigating this challenge, we have recently developed a new class of ionic LCs; the salts of amphiphilic carboxylic acids with 2-amino alcohols, or those of carboxylic acids with amphiphilic 2-amino alcohols, have a strong tendency to exhibit thermotropic LC phases. Because of the noncovalent nature of the interaction between molecules, one of the two components can easily be exchanged with, or transformed into, another molecule, without distorting the original LC architecture. In addition, both components are common organic molecules, and a variety of compounds are easily available. Taking advantage of these characteristics, we have succeeded in applying two-component LCs as chiral media for molecular recognition and reactions. This review presents an overview of our recent studies, together with notable reports related to this field.

## 1. Introduction

To mimic natural systems, chemists have long sought tailored micro-environments, which are capable of incorporating, sensing, and/or transforming organic molecules with high selectivity and efficiency. For this aim, a number of constrained micro-environments have been explored to date, including crystals [1,2,3], coordination polymers [4,5,6], zeolites [7,8,9], clays [10,11], graphite [12] and discrete hosts in homogeneous solutions [13,14,15]. Among them, crystals are regarded as one of the most successful classes. Because of their highly ordered structures, molecular events in crystalline matrices often proceed in an extremely controlled manner. In fact, solid-state hosts based on crystals, such as inclusion complexes and metal-organic frameworks, occasionally incorporate guest molecules with high selectivity [2]. In addition, such solid-state hosts potentially switch their chemical/physical properties depending on the amount and shape of incorporated guests, which is induced by the transformation of their frameworks in response to the adsorption and desorption of guests [5]. Moreover, when the components of a crystalline structure possess chemically reactive moieties, their *in situ* reactions may proceed in an ultimately selective manner, due to the pre-organized arrangement of crystalline matrices [1,3]. Owing to these unique properties, solid-state hosts based on crystalline systems would find widespread application as selectors, sensors, actuators, and reaction media, *etc*.

In spite of their prominent advantages, however, crystal-based micro-environments have not taken a leading role in current materials chemistry. Most likely, one of the most serious obstacles is the difficulty of *in situ* reactions within crystalline matrices. In general, molecular motions in crystalline systems are highly restricted to reduce the probability of reactions, and therefore, crystalline-phase reactions can proceed only when crystal packing meets topochemically stipulated demands [3]. In fact, most of the crystalline-phase reactions reported to date were found accidentally or as a result of tedious trial-and-error processes. For the same reason, *in situ* polymerization of components within crystalline matrices hardly proceeds efficiently; although this approach would solve the intrinsic drawbacks of crystalline materials, such as low mechanical strength and intolerance to solvents, *etc*.

To overcome such limitations, one of the most promising approaches is to employ liquid crystals (LCs) in place of crystals. Compared with the other mesophase aggregates, such as micelles, physical gels, and bilayers, *etc*., the structural order of LCs is generally considered to be much higher, which ranks next to that of crystals. And yet, certain mobility of molecules is ensured in LC matrix, which allows for a high probability that the *in situ* chemical reaction will proceed. Therefore, *in situ* reactions in LCs would lead to a new type of tailored micro-environment, which may realize highly efficient/selective molecular recognitions and chemical reactions. This review summarizes our recent effort to develop a new class of micro-environment based on the *in situ* reactions in LCs [16,17,18,19,20]. In relation to our studies, we also provide an overview of notable works related to LC-template reactions, from historically important reports to recent trends.

## 2. Classification of *In Situ* Reactions in Liquid Crystals

Many kinds of *in situ* reactions in LCs have been reported to date, of which potential application ranges over various fields in material science, which are not limited to the fabrication of micro-environments for molecular recognition and chemical reactions [21,22,23,24], as mentioned in Section 1. To clarify the scope of this review, this section presents the classification of *in situ* reactions, according to the number of components and the location of reactive moiety.

Traditional thermotropic LCs are composed of a single mesogen, or a mixture of a few mesogens with a similar structure. When a reactive functionality is introduced to the mesogen unit, the *in situ* reactions, both of the intra- and intermolecular reactions, would become possible. These reactions have proven to be powerful tools for scientific studies and practical applications of LCs. For example, the dimerization/polymerization of mesogen units enhance mechanical strength, stabilize LC phases, and memorize structural information [21,22]. On the other hand, intramolecular reactions, such as isomerizations, cyclizations, and rearrangements, would lead to stimuli-responsive systems [23], or serve as probes to investigate the structure/properties of LCs [24]. Although the resultant materials would find various applications, such as photo-optical/electronic devices, their self-completed structures are not suitable to accommodate exterior guest molecules.

**Figure 1:**
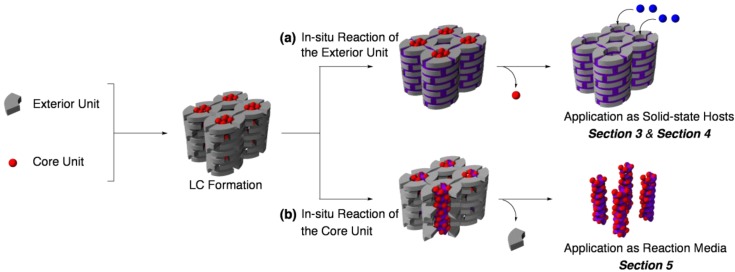
*In situ* reactions of two-component LCs at (**a**) exterior and (**b**) core units.

Compared with single-component LCs, multi-component LCs seem to provide more promising platforms for molecular recognition and chemical reactions [25,26]. Depending on the structure and compositions, multi-component LCs can be roughly classified into two categories, lyotropic LCs and supra-molecular thermotropic LCs. Here, as the simplest model of both classes of multi-component LCs, we deal with a two-component LC composed of a lipophilic exterior unit and a lipophobic core unit (Figure 1). Such an assembly might be regarded as one kind of host–guest system, because (i) these two units interact with each other via non-covalent interactions and (ii) the exterior units (hosts) take surrounding positions of the core units (guests). For the *in situ* reaction of the two-component LC, reactive functionalities can be introduced to either of the exterior and template units. As described in the following part, this choice is a determinant factor for the applicable areas of these systems.

Intermolecular reactions of the exterior parts, such as the dimerization, polymerization, and cross-linking, would “freeze-in” the whole structure of LC (Figure 1a). The most unique characteristic of the resultant material is that the core unit should be easily removed without inducing a serious loss of the LC ordered structure, owing to the noncovalent nature of the interactions between the exterior and template units, together with the covalently linked network at the exterior region. After the removal of core units, the functional groups in the exterior units lose their counterparts, which might be available as interaction sites for the next guest incorporation. As a result, the *in situ* reactions at the exterior part would provide robust, reusable porous materials, which are capable of capturing the original template units and related guests. Application of such materials is discussed in Section 3 and Section 4.

On the other hand, reactions in the core parts proceed in a quite peculiar environment, where substrates are confined in a small, constrained space (Figure 1b). Compared with the exterior part, composed of flexible units, the core part usually takes on a highly ordered structure, which seems to be an attractive micro-environment for the control of chemical reactions. Especially, the stereocontrol of intermolecular photoreactions still remains as a challenging target, generally difficult within the framework of traditional synthetic methods. Section 5 focuses our recent efforts on this aim.

## 3. Guest-Selective Molecular Sieves

By cross-linking the exterior part of multi-component LCs, new type of porous materials would be afforded, of which cavities should possess the size, shape, and electron density distributions complementary to those of the template core unit. This method is reminiscent of well-known molecular imprintings, but the pre-organized alignment of the components in LCs is expected to solve the long-standing problem in the field of traditional molecular imprinting, *i.e.*, the polyclonality of cavities [27,28,29]. Thus, the resultant materials are expected to serve as molecular sieves with guest selectivity superior to conventional molecularly imprinted polymers.

As very rare successful examples of template polymerization within organic crystals, Matsumoto *et al.* have reported on the topochemical polymerization of the salts of 1,3-diene mono- and dicarboxylic acids with primary amines [30]. The topochemical polymerization of these salts provides organic intercalation hosts with extremely high structural order, which have found unique applications [31]. Although this is one of the most ideal approaches to access rationally designed zeolite analogues, the scope of this methodology is quite narrow due to the low probability of topochemical polymerization.

### 3.1. Size recognition by cross-linked LCs

Lyotropic LCs inherently localize the ionic head-groups of the constituent amphiphiles into the interior of their aqueous channels [32]. When the head-groups are composed of functional groups with catalytic activity, the aqueous channels are expected to offer a unique environment for organic transformations. The special arrangement of the functional groups, closely packed within a small channel, would enhance their acidity/basicity, due to the changes in the dielectric constant and/or surface potential. In addition, the well-regulated size of the aqueous channels is expected to serve as size-selective catalysis.

**Figure 2:**
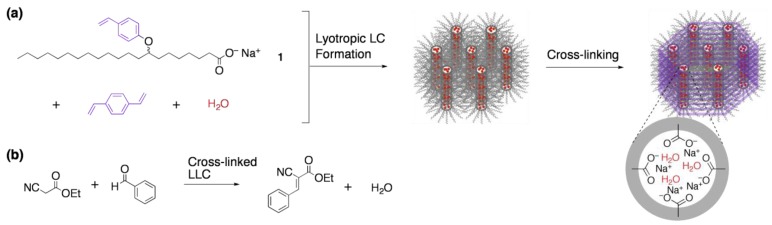
Heterogeneous catalyst based on the cross-linking of a lyotropic LC: (**a**) preparation; and (**b**) application, as a heterogeneous catalyst of the Knoevenagel condensation.

As a pioneering work in this field, Gin and co-workers have extensively studied the development of functional materials based on the cross-linking of lyotropic LCs with inverted hexagonal and bicontinuous cubic structures [33]. They developed a polymerizable amphiphile, a styryl derivative of sodium 9-octadecanoate (**1**). By mixing **1**, water, and divinylbenzene in an appropriate ratio, an inverted hexagonal lyotropic LC phase was formed, which can be cross-linked by the photopolymerization (Figure 2a). Through the cross-linking process, the original hexagonal structure was retained. The cross-linked polymer acted as an effective heterogeneous catalyst for the Knoevenagel condensation of ethyl cyanoacetate with benzaldehyde (Figure 2b). Catalytic efficiency of the cross-linked polymer was superior to that of the sodium- exchanged versions of zeolite-Y and MCM-41, which might be attributable to the enhanced basicity of the sodium carboxylate groups (p*K*_a_ = *ca.* 9) due to the confinement within the narrow aqueous channels. Although the size-selective catalysis was not explored in this report, preliminary experiments proved that the cross-linked polymer showed size exclusion ability; upon soaking the cross-linked polymer in a solution of a cationic dye, efficient incorporation took place only when the dye was smaller than the diameter of the channel (15–20 Å).

Based on the same approach, Gin and co-workers have developed various heterogeneous catalysts, by using analogous polymerizable amphiphiles bearing a head-group of, for example, sulfonic acid [34], scandium (III) sulfonate [35], and chiral imidazolidinone derivative [36], *etc*.

### 3.2. Functional group recognition by cross-linked LCs

Compared with lyotropic LC systems, one of the most prominent advantages of thermotropic LCs is their simple structure and composition. In the case of lyotropic LC systems with an inverted micelle structure, the inner space of the aqueous channel is occupied by an uncertain number of water molecules, of which precise positions are not defined and are virtually unpredictable. On the other hand, relative orientation of the components in thermotropic LCs is easy to estimate, especially when relatively strong interactions work between the components. By careful design of the components, channels complementary to the template unit would be created, which might enable us to rationally control the structure and properties of the resultant channels.

**Figure 3:**
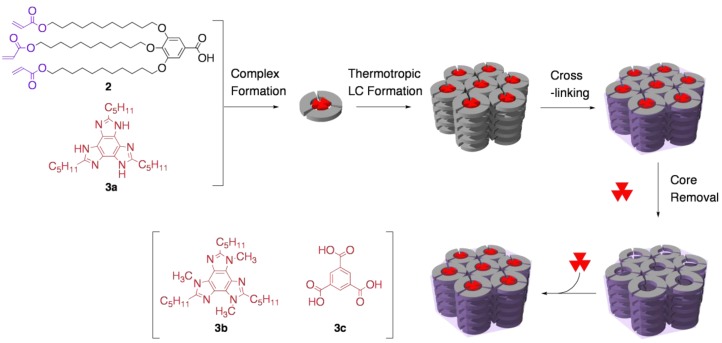
Solid-state host based on the cross-linking of a thermotropic LC: Preparation and functionality selective incorporation of guests.

As a representative example, Kim and co-workers have reported a nanoporous cross-linked polymer with hexagonal columnar channel, based on a 3:1 supramolecular complex of a polymerizable amphiphilic carboxylic acid **2** with a benzotris(imidazole) core **3a** (Figure 3a) [37]. The complex spontaneously formed a hexagonal columnar LC phase. Upon irradiation of the LC with UV light, the polymerization of the acryloyl moieties readily proceeded to give the cross-linked polymer. By the treatment of the resultant polymer with acidified methanol, *ca.* 90% of the template was extracted. Through the cross-linking and template-removal processes, the polymer maintained the same hexagonal columnar structure. Worth noting is that nitrogen gas permeability constant of the de-cored cross-linked polymer was four orders of magnitude higher than that of low-density polyethylene, which confirms the porous nature of the cross-linked polymer.

Another unique feature of the cross-linked polymer is the capability of recognizing the structural difference of guest molecules (Figure 3b). The de-cored polymer readily incorporated the original template **3a**, while other guest molecules with the right size to fit into the channel were hardly captured, such as the tri-*N*-methylated derivative of **3a** (**3b**) and 1,3,5-benzenetricarboxylic acid (**3c**). Although experimental details of the host-guest chemistry were not given, this report might be the first demonstration proving one can access shape-selective porous material through the template polymerization of LCs.

### 3.3. Chirality recognition by cross-linked LCs

The above works clearly demonstrate the potential utility of the solid-state hosts based on the *in situ* cross-linking of LCs. However, the molecular recognitions performed in these works are at the preliminary stage, where the discrimination of guest molecules with large differences in their size or polarity was achieved. A more attractive challenge is the recognition of a very subtle difference in the shape of guest molecules, such as the differentiation of diastereomers and enantiomers.

As suitable platforms to create cross-linked LCs with chirality recognition ability, we have recently developed a new class of ionic LCs; the salts of amphiphilic carboxylic acids with 2-amino alcohols have a strong tendency to exhibit thermotropic LC phases [16,18]. Considering the characteristic properties of the core unit (= 2-amino alcohol), our system would have special meaning from the following viewpoints: (i) 2-amino alcohols are one of the most easily available classes of enantiopure materials, which allow us to prepare various chiral architectures just by changing the core unit. (ii) The separation of the regio-/stereo-isomers of amino alcohols is of significant importance, because they are indispensable materials in most of the scenes of chiral technology.

**Table 1:**
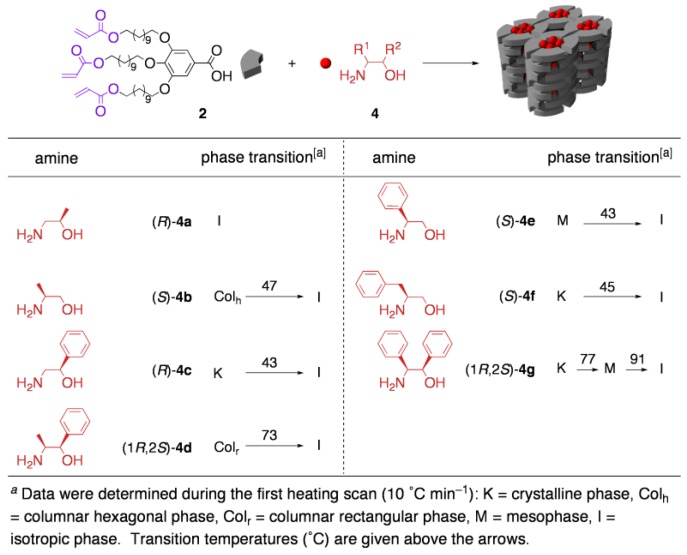
Phase transition behavior of the salts of a polymerizable carboxylic acid (**2**) with various 2-amino alcohols (**4a**–**4g**).

In order to probe the scope of this LC system, the salts of the polymerizable amphiphilic carboxylic acid **2** with various 2-amino alcohols (**4a**–**4g**) were prepared. As summarized in Table 1, four of the six salts exhibited a LC or mesophase within a wide thermal region. According to the lattice parameters and the molecular models, the number of acid–base pairs included in the layer of a cylinder was estimated; four pairs for the hexagonal columnar structure and three or four pairs for the rectangular columnar structure, respectively. When the amino and/or hydroxy group of these 2-amino alcohols were methylated, the LC phases were no longer formed or became unstable, indicating that hydrogen-bonding interactions between the carboxyl groups with the amino and hydroxy groups play an essential role for stable LC phase formation [38,39].

**Figure 4:**
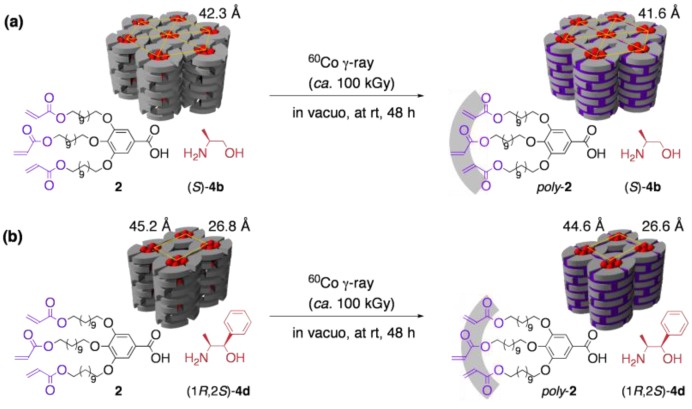
*In situ* cross-linking of two-component LCs with (**a**) hexagonal and (**b**) rectangular columnar structures.

Among these LC salts, **2**·(*S*)-**4a** (hexagonal) and **2**·(1*R*,2*S*)-**4d** (rectangular) were applied to the *in situ* polymerization. Regardless of the packing mode of the LC structure, the LC-based template polymerization proceeded successfully; upon irradiating ^60^Co γ-ray to the LC salts, the polymerization of the acryloyl groups quantitatively took place to afford cross-linked polymers, which were insoluble to most of solvents and did not melt at high temperatures. Through the cross-linking process, the original ordered structures in LCs were essentially retained (Figure 4).

In the next stage, molecular recognition ability of the resultant cross-linked materials was estimated. For this aim, the cross-linked polymer from **2**·(1*R*,2*S*)-**4d** was used, because the aromatic group in the core unit enables UV detection in HPLC analysis. Through the guest-exchanging reaction, the cross-linked polymer was found to work as a size- and shape-selective molecular sieve for amine guests. As summarized in Table 2, the topological shape of the guests was one of the most crucial factors for the efficiency of guest exchange. In the case of guests of which the C2 position was unsubstituted ((*R*)-**4a** and (*R*)-**4c**) or substituted with a relatively small group such as methyl and ethyl group ((*S*)-**4b**, (*S*)-**4h**, and (1*R*,2*S*)-**4k**), the guest exchange proceeded smoothly to achieve an incorporation of about 30%. Contrary to this, another family of guests, bearing a bulky group at the C2-position, showed much lower affinity toward the polymer (22 and 10% for **4i** and **4e**, respectively). Even in competitive guest exchanging reactions, the same tendency in guest preference was again observed, which clearly demonstrates the potential for the practical utility of the polymer as a selector for the separation of amines. For example, when an equimolar mixture of two regio-isomers ((*R*)-**4c** and (*S*)-**4e**) was applied to the guest exchanging reaction, the amount of (*R*)-**4c** incorporated in the polymer was about three times higher than that of (*S*)-**4e**.

**Table 2:**
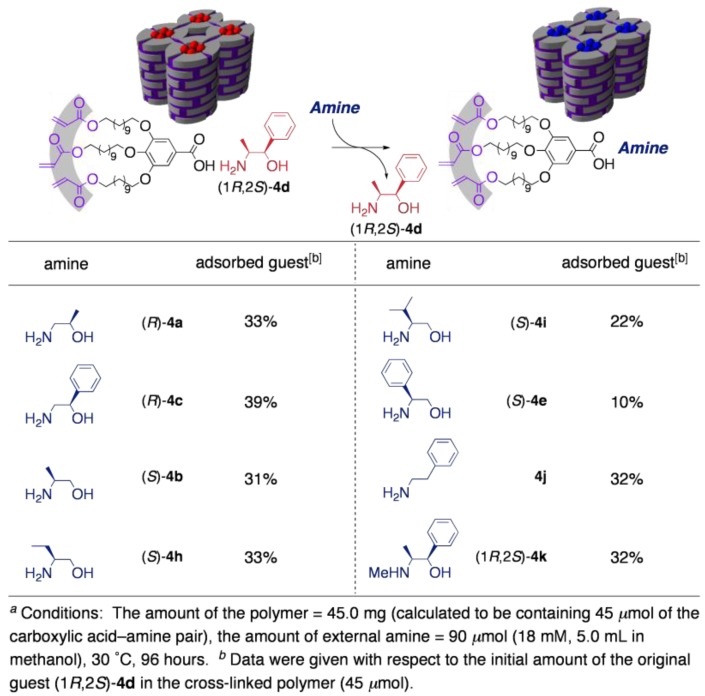
The guest exchanging reaction of (1*R*,2*S*)-**4d** (schematic shown above Table) in the cross-linked polymer with various amines.^[a]^

As a more intriguing application, performance of the cross-linked polymer as chiral selector was investigated. By using several racemic guests, the guest-exchanging reaction was conducted under competitive conditions. As a result, the cross-linked polymer proved to work as a chiral molecular sieve (Table 3). After 30–60% conversion of guest exchanging, the guest amines remaining in the exterior solution became non-racemic, indicative of the enantioselective incorporation into the cross-linked polymer. Relatively high selectivity was observed in the case of substrates possessing a bulky substituent at the C1 position (entries 2 and 3). Worth noting is that the enantiomer preferentially adsorbed was of the absolute configuration at the C1 position identical to that of the original core (1*R*,2*S*)-4d, when the guests have a substituent only at the C1 position. This observation strongly suggests that a ‘template effect’ certainly works in this system. Although the enantiomeric excesses observed here were apparently too small for practical application, worth noting is that these values were the result of only a one-batch process; the differences in the adsorption energy (*ΔΔG*) between the pairs of the enantiomers were calculated to be no less than 0.14–0.48 kcal mol^–1^. This result implies the potential utility of the present polymer as a selector for multistage separations, such as a stationary phase for chiral chromatography, where the *ΔΔG* ≥ 0.1 kcal mol^–1^ is a criterion for practical use [40].

**Table 3:**
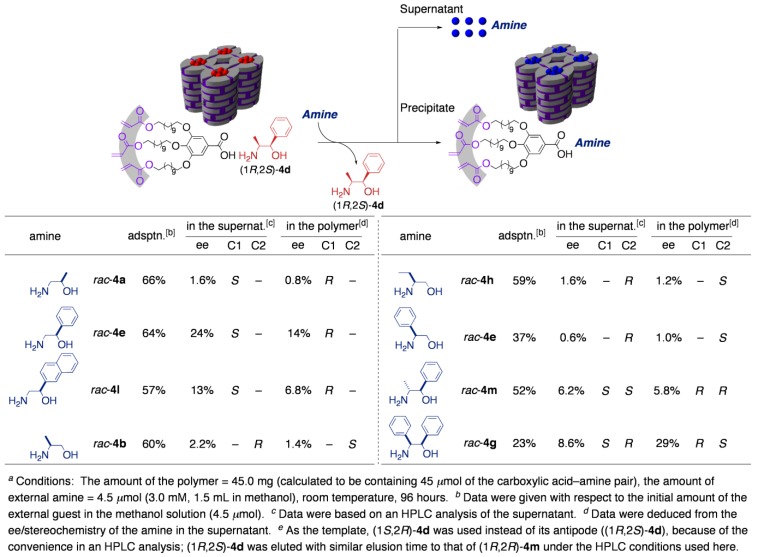
The guest-exchanging reaction of (1*R*,2*S*)-**4d** in the cross-linked polymer with various racemic amines.^[a]^

## 4. Guest-Responsive Frameworks

As described in Section 3, porous materials obtained by the cross-linking of LCs serve as selectors to incorporate guest molecules in size-, shape-, and enantio-selective manners. Considering the structures of such porous cross-linked polymers, they are expected to work as guest-responsive frameworks as well. In most of host–guest complexes in the solid state, removal of guests generates vacant spaces, which are thermodynamically unstable in general. As a result, frameworks with a flexible nature tend to change their structures in response to the guest desorption. Such a structural switching often triggers dramatic changes in the structures/properties of the whole system, which leads to the development of sensors, logical gates, and stimuli-responsive actuators. In fact, guest-responsive dynamic natures of solid-state, such as graphites [12], clays [10], and coordination polymers [5], have been extensively studied. Despite their potential application, however, these traditional solid-state hosts generally lack physical robustness, kinetic stability and processablity, because components in these systems are mainly connected by noncovalent interactions. On the other hand, cross-linking of LC components by covalent bonds would provide a new type of guest-responsive framework.

### 4.1. Relationship between LC packing mode and structural flexibility

**Figure 5:**
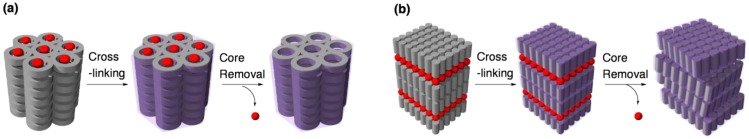
Schematic representation of the fabrication of organic zeolite analogues by the cross-linking of multi-component LCs and the following core removal: (**a**) hexagonal columnar phase and (**b**) lamellar phase.

In the case of conventional solid-state hosts, the packing mode of molecules has a large influence on the structural flexibility of the assemblies. Considering the structural homology, the same tendency is considered to exist in the case of the cross-linked LCs. Unfortunately, however, reported examples of the cross-linked LCs based on the present approach have been very limited to date, and therefore, systematic studies are still missing. In a recent report by Kishikawa *et al*., a notable prediction is provided [41]. According to their expectancy, cross-linked LCs with hexagonal columnar structures will retain the size/shape of the pore after the removal of the core, owing to the rigidity of the honeycomb structure (Figure 5a). Contrary to this, in the cases of lamellar systems, the removal of cores is likely to induce the squashing of the original structures; in these systems, the cross-linking network is likely to be made only in an intra-layer manner, while the space between the cross-linked layers is considered to be occupied only by the core units (Figure 5b).

In the report of cross-linked lyotropic LCs by Gin and co-workers, structural rigidity of hexagonal columnar and lamellar structures is briefly described [33]. They used mixtures of polymerizable amphiphile **1** and divinylbenzene, which could pack into a lamellar or hexagonal columnar structure depending on the amount of water added. For both of the assemblies, the *in situ* cross-linking was successfully conducted by the photopolymerization, where the original structures were essentially retained. To assess the stability, the resultant cross-linked polymers are extracted with dry tetrahydrofuran at reflux, retrieved by filtration, and analyzed by XRD. The cross-linked polymer with hexagonal columnar structure showed a slight decrease in overall order, while the lamellar system showed greater disorder, which was confirmed by XRD measurement. These observations are in good agreement with the above prediction by Kishikawa, *et al.*

Another supportive example is found in a system based on thermotropic LC, *i.e.*, the report by Kim *et al.* as described in Section 3.2 [37]. The cross-linked polymer with a hexagonal columnar structure, prepared from a thermotropic LC, composed of a polymerizable amiphiphilic carboxylic acid **2** with a benzotris(imidazole) core **3a**, retained its original structure even after the removal of the core units. In principal, columnar thermotropic LCs can take several packing modes of columns, such as lamellar, oblique, rectangular, tetragonal, and hexagonal structures. By using the *in situ* polymerization of these thermotropic LCs, a systematic study would be possible to correlate a molecular packing mode with structural flexibility.

### 4.2. Tuning the structural rigidity of cross-linked LCs

**Figure 6:**
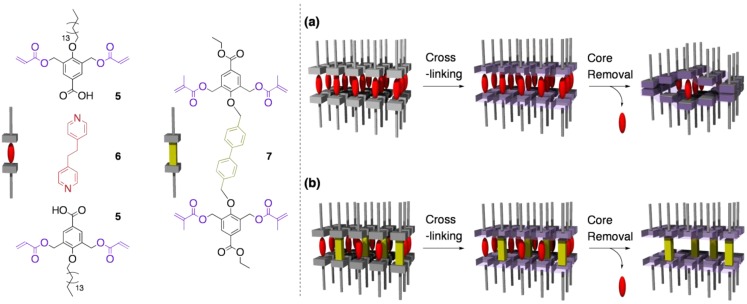
Schematic representation of the supramolecular monomer (**5**-**6**-**5**), the pillar molecule (**7**), and the processes of constructing ordered polymers from **5**-**6**-**5** and **7**.

According to the assumption of Kishikawa *et al*., solid-state hosts based on the cross-linking of lamellar LCs are intrinsically intolerant to the core removal. In the same report, they proposed an intriguing new methodology for controlling the rigidity of such architectures with lamellar structures [41]. Their approach involves the introduction of “nanopillars” between the layers, which was expected to support the lamellar structure after the removal of the core units (Figure 6).

As a fundamental skeleton, they newly developed a two-component thermotropic LC with a lamellar structure (smectic A), based on the 2:1 supramolecular complex of a carboxylic acid bearing two acryloyl groups (**5**) with a dipyridyl core (**6**). A pillar molecule was also designed as a rodlike molecule possessing polymerizable units (**7**). When 5 and 10 mol% of the pillar unit **7** was doped in the two-component LC composed of **5** and **6**, the resultant mixtures again showed the same lamellar phases. The three LC systems, containing the pillar unit **7** at the molar ratio of 0, 5, and 10%, respectively, were successfully cross-linked by the photopolymerization, where the original lamellar structures were maintained.

By treating of the three cross-linked polymers with diluted HCl, the core unit **6** was gradually extracted into the supernatant. Worth noting is that the efficiency of the extraction changed dramatically depending on the amount of doped **7**. In the case of the cross-linked polymer composed of only **5** and **6**, no more than 50% of **6** could be removed. In sharp contrast, the cross-linked polymers doped with 5 and 10 mol% of **7** could release 77 and 100% of **6**, respectively. Such a large difference in the efficiency of the core removal might be attributable to the structural changes during the core removal process. In the absence of the pillar unit, partial removal of the cores from the whole structure of the cross-linked polymer would give a squashed layer structure, preventing the removal of the remaining core units from the inner spaces. On the other hand, the cross-linked polymer doped with the pillar **7** was likely to maintain the layered structure during core removal, so that the channels for the efficient mass transfer would be kept throughout the process. A preliminary XRD analysis of the pillared polymer suggested that the lamellar structure was retained after core removal.

### 4.3. Reversible structural switching of cross-linked LCs

**Figure 7:**
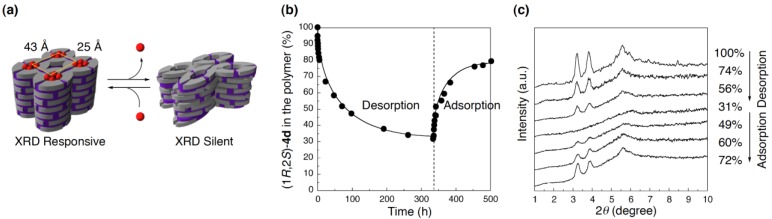
Desorption/adsorption of the template (1*R*,2*S*)-**4d** from/to the cross-linked polymer: (**a**) Schematic representation of the structural switching; (**b**) Time course of the desorption/adsorption monitored by HPLC; and (**c**) XRD profiles of the cross-linked polymer.

As described in Section 3.3, we developed a cross-linked polymer with rectangular columnar structure (space group; *P*2m), which was prepared from the LC salt of an polymerizable amphiphilic carboxylic acid **2** with an enantiopure 2-amino alcohol (1*R*,2*S*)-**4d** [16,18]. Through the studies on host–guest chemistry of the cross-linked polymer, we unexpectedly found that our cross-linked polymer expressed a dynamic behavior in response to the desorption and adsorption of guest molecules (Figure 7a) [17].

The cross-linked polymers as prepared were used as the starting material, of which binding sites were fully occupied with the original core (1*R*,2*S*)-**4d**. Upon soaking the cross-linked polymer in a methanolic HCl solution, (1*R*,2*S*)-**4d** in the cross-linked polymer was leached out to the supernatant, and an equilibrium was attained when 69% of (1*R*,2*S*)-**4d** was desorbed from the cross-linked polymer (Figure 7b). Through the removal of the template, a dramatic structural alteration was induced diminishing the intensities of the XRD reflections characteristic of the rectangular columnar structure. At the final equilibrium stage, the three diffractions became undetectable (Figure 7c).

Re-adsorption of the template to the de-cored polymer was then performed, by soaking the de-cored polymer in a methanol solution of the original core (1*R*,2*S*)-**4d**. At an equilibrium state, total amount of (1*R*,2*S*)-**4d** in the cross-linked polymer became 79% with respect to the carboxylic acid units in the cross-linked polymer (Figure 7b). To our surprise, the re-adsorption of (1*R*,2*S*)-**4d** to the cross-linked polymer undoubtedly triggered the reconstitution of the ordered rectangular columnar structure, of which the lattice parameters were essentially identical to those of the original polymer before the desorption of (1*R*,2*S*)-**4d**; three characteristic diffractions emerged even when only 49% of the carboxylic acid units in the cross-linked polymer participated in the salt-pair formation with (1*R*,2*S*)-**4d**, and their intensities became stronger as the re-adsorption proceeded (Figure 7c). Any other diffraction was not observed through the re-adsorption process, which strongly suggests that there is little possibility of the transformation of the cross-linked polymer into another ordered structure.

### 4.4. Guest-selective structural switching of cross-linked LCs

**Figure 8:**
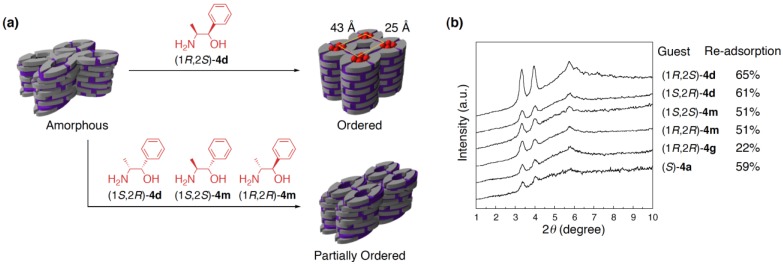
Guest-selective structural switching of the cross-linked polymer: (**a**) Schematic representation; and (**b**) XRD profiles of the cross-linked polymers obtained by the reconstitution of the apo-polymer with various amino alcohols.

Our cross-linked polymer, prepared from the LC salt of **2** with (1*R*,2*S*)-**4d**, is capable of incorporating amines other than the original core (1*R*,2*S*)-**4d**, as described in Section 3.3. In the guest-binding process, guest molecules with a more similar structure to the original core showed higher binding affinity to the cross-linked polymer. This observation clearly indicates that some structural information of the original core was certainly “imprinted” in the cross-linked polymer. Such a template effect would also have a large influence on the guest-responsive transformation behavior of the cross-linked polymer (Figure 8a) [17].

To prove this concept, several 2-amino alcohols with similar structures to that of the original template, (*S*)-**4b**, (1*S*,2*R*)-**4d**), (1*S*,2*S*)-**4m**, and (1*R*,2*R*)-**4m**, were employed as triggers to induce the structural change of the de-cored polymer. In every case of these 2-amino alcohols, the polymer exhibited XRD profiles characteristic of the original rectangular columnar structure (Figure 8b). Worth noting is that there was significant difference in reconstitution ability between the original core (1*R*,2*S*)-**4d** and its enantio/diastereo isomers (1*S*,2*R*)-**4d**, (1*S*,2*S*)-**4m**, and (1*R*,2*R*)-**4m**; although the amounts of the re-adsorbed guests at an equilibrium state were almost identical in all of the four cases (51–65%), the intensities of the diffractions of the polymers, reconstituted with these three new guests, were about half that of the corresponding diffractions observed in the reconstitution with (1*R*,2*S*)-**4d** (Figure 8b).

These observations suggest that the three-dimensional cross-linking might cause considerable structural constraint on the resultant polymer, which remained even after the removal of the original template. As a result, the de-cored polymer had a strong tendency to take the rectangular columnar structure regardless of the structure of an incorporated guest, and yet the polymer host possessed an ability to sense the subtle structural difference between the diastereo/enantio isomers so that the “best fitting” was achieved only when the original core was used as the guest.

## 5. Tailored Reaction Media

Constrained reaction environments have attracted continuous attention as one of the simplest models of biological reaction systems. In addition, media-controlled reactions might establish a practically useful methodology, which play a complementary role to traditional organic synthesis in homogeneous media. Particularly, enantiocontrol of intermolecular photochemical reactions has remained as an unexplored issue in the framework of homogeneous reaction systems. Despite such significance, however, the idea of media-controlled reactions has an inherent limitation, a “trade-off” between selectivity and reactivity. As described in the introduction, extremely ordered media as represented by crystalline reaction systems occasionally realize almost perfect reaction control, but in most of the cases, the severe restriction on molecular motions fatally reduce the probability of reactions [1,3]. Whereas, mesophase aggregates, such as micelles [42,43], physical gels [44,45,46], and bilayers [47,48], *etc*. are generally unable to realize ideal selectivity, although their relatively loose structures are promising for the promotion of the *in situ* reaction. In attempts to overcome these problems, several groups have employed LCs as media to control chemical reactions [24,49,50]; among mesophase aggregates, thermotropic LCs are regarded as one of the most structurally ordered classes, which should be advantageous for the control of organic transformations.

### 5.1. Asymmetric synthesis in cholesteric LCs

Cholesteric LCs have been extensively studied in the 1970s as media for asymmetric reactions. Apparently, their helical structure seems to be quite promising for the chirality induction in organic transformations. Saeva *et al.* reported that the Claisen rearrangement of methylallyl *p*-tolyl ether (**9**) proceeded in an enantiocontrolled manner in a cholesteric LC mesophase formed by cholesteryl *p*-nitrobenzoate (**8a**) to afford the optically active 2-(α-methylallyl)-4-methylphenol (**10**) [51]. When the reaction was conducted at an isotropic phase, the optical activity of the rearrangement product **10** was not observed, which suggests that the chirality induction was mainly due to the formation of the chiral mesophase rather than the diastereomeric interaction between **8a** and **9**. Unfortunately, enantiomeric excess of the rearrangement product was not clearly given in this report.

**Figure 9:**
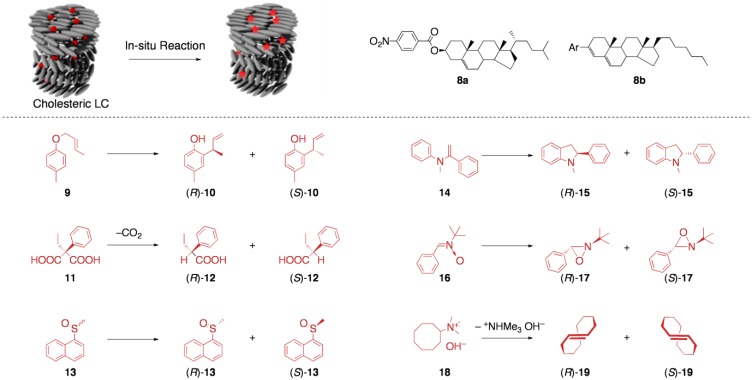
Asymmetric synthesis in cholesteric LCs.

Another earlier example is the report by Verbit *et al.*; they found that the decarboxylation of ethylphenylmalonic acid (**11**) within the cholesteric LC mesophase composed of cholesteryl benzoate (**8b**) gave phenylbutyric acid (**12**) in an *R*-enriched form (18% *ee*) [52]. As in the case of the report by Saeva *et al.*, they also suggested that the helical macrostructure of the cholesteric mesophase rather than the local asymmetry of the steroid system was the determinant factor for the asymmetric transformation. They further postulated that the same reaction conducted in another cholesteric LC of the opposite macrostructural handedness afforded *S*-enriched **12**.

After these works, reaction media based on cholesteric LCs have been applied to other reactions, such as the deracemization of sulfoxyde (**13**), the photocyclization of α-(*N*-methylanilino)styrene (**14**) to an indoline (**15**), the photocyclization of a nitrone (**16**) to an oxazolidine (**17**), and the formation of *trans*-cyclooctene (**18**) via the Hofmann elimination of an ammonium derivative (**19**) [53,54,55]. Overall, most of these attempts have resulted in unsatisfactory selectivity, especially in terms of enantioselectivity (up to 20% *ee*), probably due to the following reasons: (i) The environment of photoreactive substrates is not necessarily “monoclonal” in these LC media, because the substrates are just mixed with chiral mesogenic components which weakly interact with them. (ii) In the attempts of enantio-control by cholesteric LC media, their helical structures (from 100 nm to μm pitch) seem to be too huge to have a significant effect on events at a molecular level. Thus, LC-media induced asymmetric reaction has still remained as a challenging target in this field. On the other hand, LC reaction media have proven to be a powerful tool to fabricate mesoscaled huge architectures.

### 5.2. Asymmetric transformation of organic molecules in lyotropic LCs

**Figure 10:**
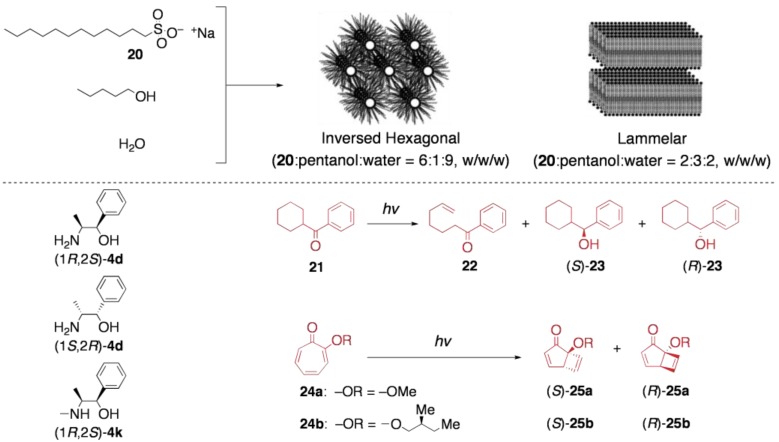
Asymmetric synthesis in lyotropic LCs.

Contrary to thermotropic LC systems, lyotropic ones have rarely been explored as media for asymmetric reactions. Recently, Wu and Tung, *et al.* have reported on the asymmetric photoreactions in lyotropic LC reaction media. They employed lyotropic LCs based on the mixture of sodium dodecyl sulfonate (**20**), 1-pentanol, and water, which were doped with substrates and chiral inducers [56,57]. As a general feature of lyotropic LCs, two types of LC structures could form from the same constituents by changing the composition; with the ratio of **20**/pentanol/water being 2:3:2 (w/w/w), a lamellar LC was yielded, while the weight ratio of 6:1:9 gave a hexagonal columnar LC (Figure 10).

In these lyotropic LCs, the photochemical transformations of cyclohexyl phenyl ketone (**22**) were conducted. Principally, the photoirradiation of **21** can lead to the intramolecular hydrogen abstraction product 1-phenyl-hept-6-en-1-one **22**. Additionally, in the presence of appropriate electron donors, the intermolecular reduction also proceeds to give a chiral product, *α*-cyclohexyl benzyl alcohol **23**. A systematic study on the product distribution revealed that these lyotropic LCs have a strong propensity to enhance the intermolecular reduction, where the ratio of **23** to **22** was 10–200 times larger than that observed in isotropic reaction systems. When optically active electron donors, such as (1*R*,2*S*)-**4d**, (1*S*,2*R*)-**4d**, and (1*R*,2*S*)-**4k** were added to these lyotropic LC systems (7.0 eq. to **22**), the reduction product **23** was obtained in nonracemic forms. Unfortunately, however, the enantiomeric excess was at an unsatisfactory level (up to 5% *ee*).

By using the same lyotropic LC media, Wu and Tung, *et al.* demonstrated the enantiocontrolled photoelectrocyclization of tropolone ethers (**24a** and **24b**) in the presence of (1*S*,2*R*)-**4d** or (1*R*,2*S*)-**4d** as a chiral inducer [57]. The hexagonal LC was found to significantly enhance the influence of chiral inducers during the cyclization (up to 40% *ee* for **24a** and 35% *de* for **24b**), while the selectivities achieved in the lamellar LC was considerably lower than those in the hexagonal LC (up to 10% *ee* for **24a** and 6% *de* for **24b**). Worth noting is that relatively high selectivities were realized although the amount of the chiral inducer was only seven times of the amount of the substrate **24**.

### 5.3. Asymmetric transformation of organic molecules in two-component thermotropic LCs

**Figure 11:**
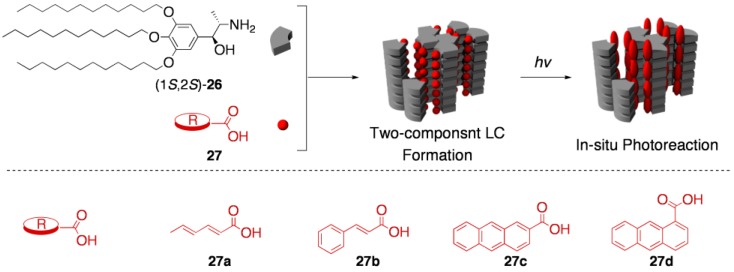
Schematic representation of the photoreaction within two-component LC media.

As described in Section 3 and Section 4, several salts of amphiphilic carboxylic acids with 2-amino alcohols were found to exist as thermotropic liquid crystals in wide thermal ranges [16,18]. Likewise, the opposite combinations, *i.e.*, the salts of amphiphilic 2-amino alcohols with carboxylic acids, were also anticipated to exhibit liquid-crystalline phases. If photoreactive carboxylic acids are employed as a component of the latter combination, photoreactions in liquid-crystalline matrices might be easily conducted just by the photoirradiation of the liquid-crystalline salts. Compared with traditional LC media, such a system seems to have obvious advantages in the following aspects: (i) The relative orientation of the substrate (carboxylic acid) molecules is considered to be well defined, because the substrate itself is the component of the LC. (ii) Every photoreactive molecule intimately interacts with a chiral source 2-amino alcohol by hydrogen-bonding interaction and salt-pair formation. (iii) Owing to the availability of various photoreactive carboxylic acids, as well as the noncovalency of interaction between the two components, such 2-amino alcohol units would offer a special environment to various substrates/reactions. As the first successful example of this approach, we recently reported that the photodimerization of 2-anthracenecarboxilic acids generated a satisfactory yield with unprecedented high enantioselectivity, by using a two component LC matrix as the reaction medium (Figure 11) [19,20].

The amphiphilic 2-amino alcohol to construct the two-component LC, (1*S*,2*S*)-**26**, was synthesized from an (*S*)-alanine derivative in a stereopure form. The salts of (1*S*,2*S*)-**26** with various carboxylic acids were prepared by mixing equimolar amounts of the units, and the mesomorphic behavior of the resultant salts was studied. As we had expected, (1*S*,2*S*)-**26** was capable of forming liquid-crystalline salts with a variety of photoreactive carboxylic acids, including sorbic acid (**27a**), cinnamic acid (**27b**), 2-anthracenecarboxylic acid (**27c**), and 1-anthracenecarboxylic acid (**27d**). Although all of the LC salts are principally applicable to the *in situ* photoreactions, the photoinduced [4+4] cycloaddition of 2-anthracenecarboxylic acids (**27c**) was chosen as our initial target because of its simple reaction course, high quantum yield, and ease for product analysis. It still remains a challenge to control the regio- and stereochemistries of the photodimerization of **27c**; the photodimerization in principle gives rise to no less than four configurational isomers (*head-to-head*/*head-to-tail* isomers with *syn*/*anti* isomerism, denoted as *syn*^HH^, *anti*^HH^, *syn*^HT^, and *anti*^HT^), two isomers of which consist of a pair of *C*_2_-enantiomers, respectively ((*R*)/(*S*)-*anti*^HH^ and (*R*)/(*S*)-*syn*^HT^) [58].

**Table 4:**
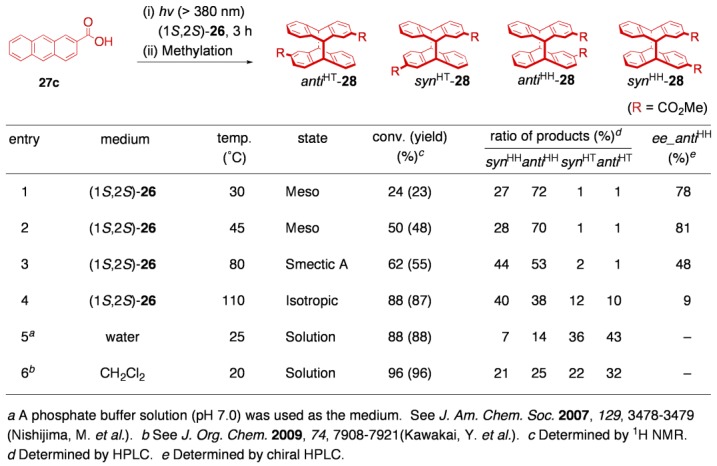
Photodimerization of 2-anthracenecarboxylic acid **27c**.

**Figure 12:**
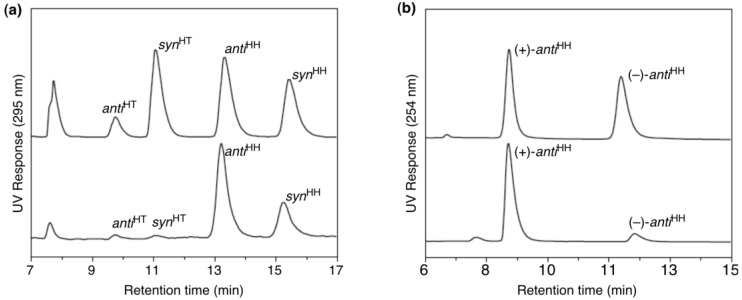
HPLC estimation of the isomer distribution of the photodimer esters **28**. (**a**) An authentic mixture of the four isomers of **28** (top) and a mixture obtained from photo-irradiated **27c**·(1*S*,2*S*)-**26** (bottom). (**b**) An authentic racemate of *anti*^HH^-**28** (top) and *anti*^HH^-**28** obtained from photo-irradiated **27c**·(1*S*,2*S*)-**26** (bottom).

The salt (1*S,*2*S*)-**26**·**27c** exhibited two kinds of thermotropic LC phases (Meso and Smectic A phases) in the cooling process from the isotropic melt. Quite interestingly, the LC matrices provided by (1*S*,2*S*)-**26** showed an excellent capability of controlling the photodimerization of **27c** (Table 4 and Figure 12a). Thus, both LC phases realized excellent regioselectivity to yield the HH dimers exclusively (HH:HT = 97:3–98:2). The selectivity was against the usual tendency (HT > HH) governed by the relative stability of the products, where the HH dimers are less stable than the HT dimers due to the electrostatic repulsion between the two carboxylate moieties, as was reported. As far as we are aware, the HH/HT ratios achieved here are the highest level in this kind of bimolecular photoreactions. Stereoselectivity in terms of *syn*^HH^/*anti*^HH^ ratio revealed a striking difference between the two LC phases. Meso phase afforded the *anti*^HH^ dimer as the main product (*syn*^HH^:*anti*^HH^ = 27:73–29:71), whereas Smectic A phase almost equally yielded the two diastereomers (*syn*^HH^:*anti*^HH^ = 45:55). Moreover, the liquid-crystalline media were found to offer a reaction environment with an excellent chirality-induction ability. Particularly, the photodimerization performed in Smectic A phase afforded the *anti*^HH^ dimer with unexpectedly high enantioselectivity (up to 81% *ee*, Figure 12b). The present outstanding selectivity is not likely to be a simple chirality transfer within the discrete salt pair, because the isotropic phase attained insufficient selectivity (9% *ee*). To the best of our knowledge, the reaction conducted in Meso phase is the first successful asymmetric synthesis induced by a chiral liquid crystal.

## 6. Conclusions

We have reviewed new aspects of template reactions in multi-component LCs. Particularly, our recent studies have featured which demonstrate the potential utility of the ionic LCs, composed of the salts of carboxylic acids with 2-amino alcohols salts, as chiral media for molecular recognition and reactions. Owing to the characteristic properties of the ionic LCs, such as ordered molecular alignment, proper mobility of molecules, and the noncovalent nature of the interactions between components, template reactions within the LC matrices proceeded efficiently. As a result, highly efficient chiral recognition, chiral sensing, and asymmetric transformation have been realized. In principle, the same strategy is applicable to other supramolecular aggregates including gels, micelles, monolayers, and bilayers, which would further enhance the expediency of chemistry in tailored media.
